# Mass spectrometry data on specialized metabolome of medicinal plants used in East Asian traditional medicine

**DOI:** 10.1038/s41597-022-01662-2

**Published:** 2022-08-27

**Authors:** Kyo Bin Kang, Eunah Jeong, Seungju Son, Eunjin Lee, Seungjin Lee, Seong Yeon Choi, Hyun Woo Kim, Heejung Yang, Sang Hee Shim

**Affiliations:** 1 Research Institute of Pharmaceutical Sciences, College of Pharmacy, Sookmyung Women’s University, Seoul, 04310 Korea; 2 Natural Products Research Institute, College of Pharmacy, Seoul National University, Seoul, 08826 Korea; 3 College of Pharmacy, Kangwon National University, Chuncheon, 24341 Korea; 4 College of Pharmacy and Integrated Research Institute for Drug Development, Dongguk University, Goyang, 10326 Korea

**Keywords:** Mass spectrometry, Pharmaceutics, Secondary metabolism

## Abstract

Traditional East Asian medicine not only serves as a potential source of drug discovery, but also plays an important role in the healthcare systems of Korea, China, and Japan. Tandem mass spectrometry (MS/MS)-based untargeted metabolomics is a key methodology for high-throughput analysis of the complex chemical compositions of medicinal plants used in traditional East Asian medicine. This Data Descriptor documents the deposition to a public repository of a re-analyzable raw LC-MS/MS dataset of 337 medicinal plants listed in the Korean Pharmacopeia, in addition to a reference spectral library of 223 phytochemicals isolated from medicinal plants. Enhanced by recently developed repository-level data analysis pipelines, this information can serve as a reference dataset for MS/MS-based untargeted metabolomic analysis of plant specialized metabolites.

## Abstract (summary)

**Table:** 

Measurement(s)	Metabolomics
Technology Type(s)	high-performance liquid chromatography-electrospray ionisation tandem mass spectrometry
Sample Characteristic - Organism	Embryophyta

## Background & Summary

Most cultures worldwide use plants to treat diseases. The integration of experimental knowledge on medicinal plant usage with theories or beliefs about health and illness is termed traditional medicine. Traditional East Asian traditional medicine is known to have originated approximately 3,000 years ago in China. It was introduced to Korea and Japan from China in the 6th century, with Buddhism and Chinese culture^1^. Since then, it has been widely used following a long history. The detailed practices are not exactly same in three countries due to several reasons, for example, the usage of different species in the same genus due to the different climate; however, they have strongly influenced each other. Traditional East Asian medicine still plays an essential role in public health care in East Asian countries; currently, standardized herbal formulae are manufactured by pharmaceutical companies and used as parts of the modern medical systems in Korea, China, and Japan. Medicinal plants used in traditional medicine are one of the most important sources of drug discovery, where artemisinin, an antimalarial agent from *Artemisia annua*, is the most representative case.

High-throughput analysis of samples with complex chemical compositions plays a key role in the investigation and modernization of traditional East Asian medicine. Tandem mass spectrometry (MS/MS), especially in combination with liquid chromatography (LC), is the analytical method most commonly used to analyze medicinal plants^2,3^. MS/MS-based untargeted metabolomics has been used to assess the quality of medicinal plants and related dietary and pharmaceutical products, and has also been utilized in structure-based bioactive compound discovery^4–7^. Despite the increased use of this technique, only a few reliable and controlled datasets of MS/MS data for medicinal plants have been deposited in public repositories. With the expansion of untargeted metabolomics to multiple fields, the importance of publicly available data is increasing. The successive launches of MASST and ReDU symbolize the increasing need for public datasets in MS/MS-based untargeted metabolomics research^8,9^. MASST enables the search for a single spectrum by comparison with all publicly available raw data, whereas ReDU enables the reuse of deposited datasets for repository-level analyses or co-analysis with the user’s own experimental data.

**Fig. 1:**
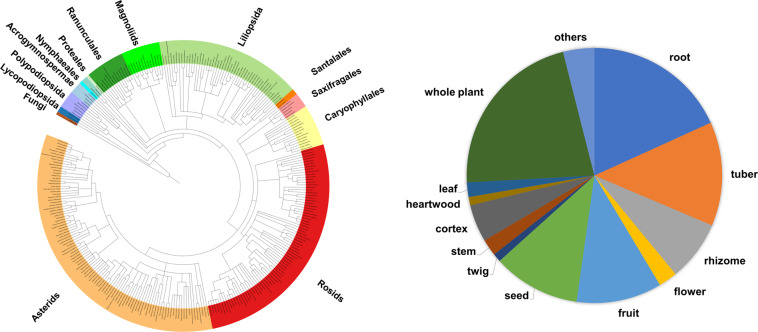
Taxonomic coverage (left) and used plant parts (right; ‘others’ includes calyx, carpel, gall, husk, peel, pericarp, pollen, and resin) of the 337 medicinal plant samples. The phylogenetic tree was constructed using the PhyloT phylogenetic tree generator, based on NCBI taxonomy^39^. Taxa of different levels are marked in the phylogenetic tree to enhance readability.

This Data Descriptor documents publicly available and re-analyzable raw LC-MS/MS dataset of 337 medicinal plants on the MassIVE raw data repository, which is linked with the Global Natural Product Social Molecular Networking platform (GNPS, https://gnps.ucsd.edu)^10^. This dataset is referred to as the KP337 dataset in this Data Descriptor, as most of the medicinal plants in the dataset are listed in the Korean Pharmacopeia (KP). The data do not cover the entire set of medicinal plants enlisted in KP, but cover most of the commonly used plants. The taxonomic coverage of the plants and plant parts used in Traditional East Asian medicine is summarized in Fig. 1. The KP337 dataset consists of raw LC-MS/MS data acquired in both positive and negative ion mode, and metadata formatted for compatibility with ReDU^9^. Thus, this dataset enables data re-usage, such as comparative analysis or propagation of spectral annotation based on spectral similarity^11^. Recently, a part of this dataset, specifically relating to various flavonoid *C*-glycosides, was utilized to establish the GNPS nearest neighbor suspect spectral library^12^. This case demonstrates the applicability of public datasets for propagation of spectral annotations. The KP337 dataset can also be applied to a MASST search of MS/MS spectra, which suggests the possible occurrence of queried molecules in medicinal plants. In natural product discovery projects, known compounds are often ignored and not reported. However, novel occurrences of known chemicals can provide insights into the medicinal or biological properties of medicinal plants, where the present dataset can contribute to such findings via MASST. Additionally, the occurrence data of known or unknown chemicals can enhance reference data-driven analysis, which was suggested as an alternative workflow for MS/MS-based untargeted metabolomics^13^.

**Fig. 2:**
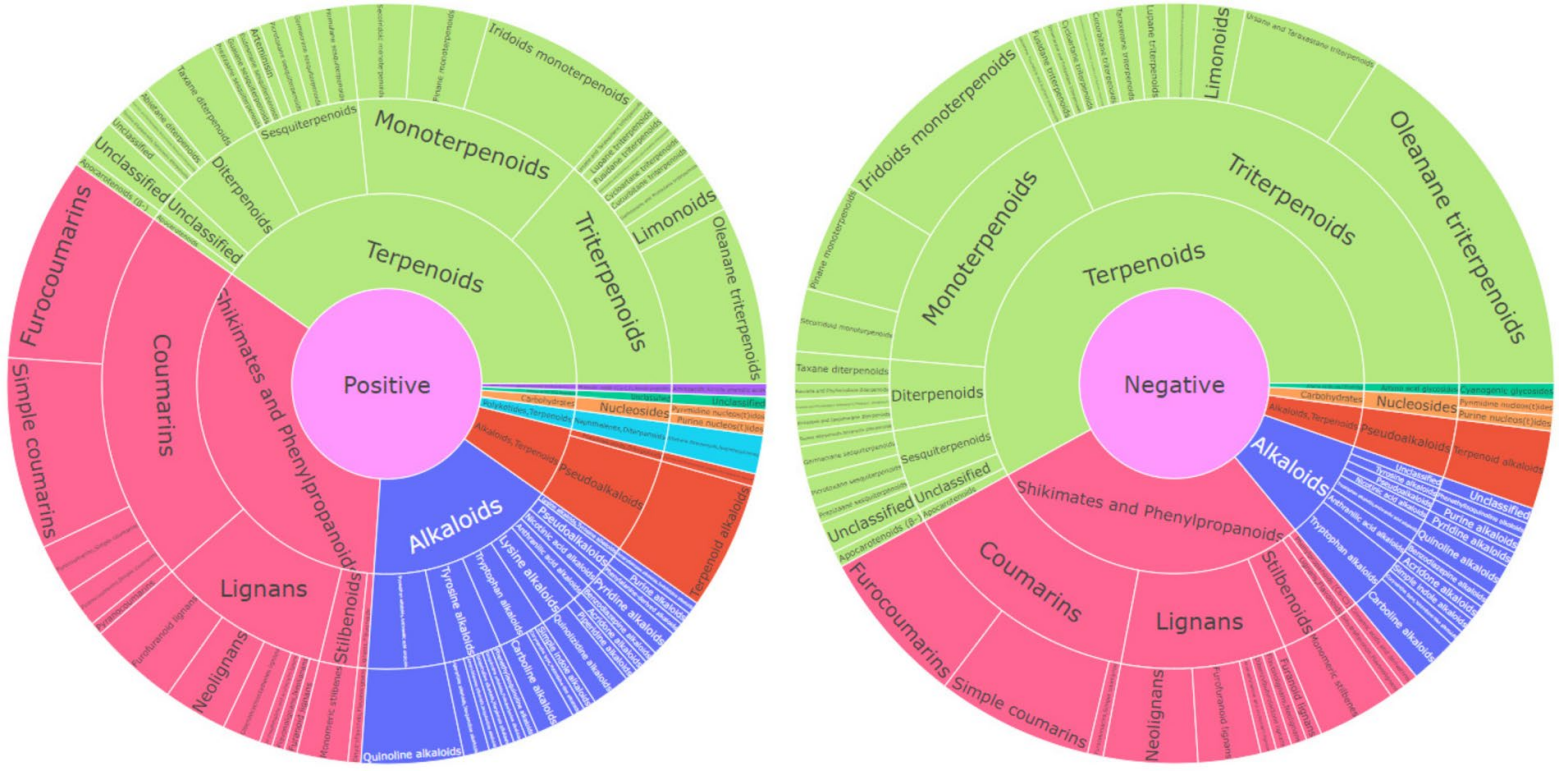
Chemical ontology of phytochemicals giving rise to positive (left) and negative (right) ion mode spectra.

We also report our efforts to establish a MS/MS spectral library of bioactive compounds obtained from medicinal plants that are used in traditional East Asian medicine. Although many phytochemicals have been previously found in medicinal plants, most of them vary in the historical collections of natural product chemistry laboratories and their MS/MS spectra have not been reported. Benchmarking recent efforts leading to the monoterpene indole alkaloid database (MIADB), and isoquinoline alkaloids and other annonaceous metabolites database (IQAMDB), which are spectral libraries built with compounds from historical collections of various natural product chemistry laboratories^14,15^, we established an MS/MS spectral library using 223 pure phytochemicals obtained from the legacy compound library of the Natural Products Research Institute, Seoul National University (SSK Legacy Library, named after Sam Sik Kang, who compiled the library over the course of 30 years). MS/MS spectra were acquired for all ionized molecules in positive (ESI+) and negative (ESI−) ion modes, which yielded 184 positive and 152 negative ion mode spectra. Compounds with low ionization efficiencies in each ionization mode were excluded. This spectral library will accelerate the annotation of phytochemicals in future metabolomic studies of medicinal plants. The chemical ontology of the phytochemicals included in the spectral library was estimated using the NPClassifier^16^ and is summarized in Fig. 2.

## Methods

### Sample preparation

The methanolic extracts of 337 medicinal plants were acquired from the Korea Plant Extract Bank of the Korea Research Institute of Bioscience and Biotechnology (Cheongju, Korea), where authentic samples were extracted with MeOH via sonication for 3 h. The extracts were dissolved in MeOH at 2 mg/mL for LC-MS/MS analysis. The collection of 223 compounds previously isolated from various medicinal plants by Prof. Sam Sik Kang (Seoul National University) has been maintained in Sang Hee Shim’s laboratory (Seoul National University), coupled with NMR spectra for structural identification. The compounds were dissolved in 50% aqueous MeOH at 100 μg/mL concentration for LC-MS/MS analysis.

### LC-MS/MS data acquisition

To compile the plant extract dataset, LC-MS/MS data were acquired using a Waters Acquity UPLC system (Waters Co., Milford, MA, USA) coupled to a Xevo G2 Q-TOF mass spectrometer (Waters MS Technologies, Manchester, UK) equipped with an electrospray ionization interface (ESI). To compose the spectral library, LC-MS/MS data were acquired using a Waters Acquity I-Class UPLC system linked to a Waters VION IMS Q-TOF MS equipped with an ESI interface. Chromatographic separation was performed using a Waters BEH C_18_ column (50 × 2.1 mm, 1.7 μm), which was eluted with a mixture of water (solvent A) and MeCN (solvent B), both acidified with 0.1% formic acid, at a flow rate of 0.3 mL/min, with a linear gradient of 5−95% B (0−14 min) followed by 3 min washing with 100% B and 3 min reconditioning with 5% B successively. The samples (2.0 μL injection volume) were analyzed in data-dependent acquisition (DDA) mode consisting of full MS survey scans in the *m/z* 100−2000 Da range (scan time: 150 ms) followed by MS/MS scans for the three most intense ions (*m/z* 100−2000 Da; scan time: 100 ms). The collision energy gradient was set as 20−100 eV. Protonated and deprotonated ions of leucine enkephalin (*m/z* 556.2771 [M + H]^+^ in the positive ion mode and *m/z* 554.2615 [M − H]^−^ in the negative ion mode, respectively) were measured in every 0.1 min as the lock mass to ensure mass accuracy and reproducibility.

### Public dataset deposition

All the raw LC-MS/MS data files were converted from the Waters.Raw format to the open-sourced.mzML format using the MSConvert tool of ProteoWizard^17^. Sample metadata were prepared to ensure compatibility with ReDU. Additional sample information which are not included in the ReDu format, such as the plant part analyzed and taxonomic ontology, are included in additional metadata files.

### Molecular networking analysis

Molecular networks were created using the classical molecular networking workflow on the GNPS web platform^10^. The networks were then created, where edges were filtered to have a cosine score above 0.65 and more than 5 matched peaks, with the precursor and fragment ion mass tolerance of 0.02 Da. The library spectra were searched to find annotation with the same score and matched peaks.

The molecular network with the positive ion mode data can be accessed via: https://gnps.ucsd.edu/ProteoSAFe/status.jsp?task=94bd6547c84341ddaaff2e4599247871

The positive ion mode MolNetEnhancer network is accessible via: https://gnps.ucsd.edu/ProteoSAFe/status.jsp?task=f896fc4740694c3fa8308dabfd2ff3c3.

The molecular network with the negative ion mode data can be accessed via: https://gnps.ucsd.edu/ProteoSAFe/status.jsp?task=d4004a9da4c84ed2b509a83813ebbea1.

The negative ion mode MolNetEnhancer network is accessible via: https://gnps.ucsd.edu/ProteoSAFe/status.jsp?task=301d52ce05244646824e6a96f97990bc.

### Spectral library constitution

The acquired LC-MS/MS files were opened with Waters UNIFI software. The list of MS features was automatically degenerated by UNIFI. MS features with hypothetical *m/z* values corresponding to commonly observed adducts ([M + H]^+^, [M + Na]^+^, [2 M + H]^2+^,[M−H]^−^, [M + HCOOH−H]^−^, and [2 M−H]^−^) were found, after which the MS/MS spectra were manually examined. The MS/MS scans related to the standard compounds were exported as.mgf files. The metadata used to establish the spectral library, including SMILES and InChI identifiers of the structures, is provided as Supplementary Table 1.

## Data Records

Raw LC-MS/MS data of the 337 medicinal plants are accessible via MassIVE (https://massive.ucsd.edu) with the accession number MSV000086161^18^. All the data can be re- or co-analyzed via ReDU^9^. MS/MS spectra of the 223 phytochemicals (184 in positive ion mode and 152 in negative ion mode) from Dr. Sam Sik Kang’s legacy chemical library have unique accession numbers from CCMSLIB00010007697 to CCMSLIB00010008032 in the spectral library of GNPS^10^, which is accessible via: https://gnps.ucsd.edu/ProteoSAFe/gnpslibrary.jsp?library=GNPS-SAM-SIK-KANG-LEGACY-LIBRARY).

## Technical Validation

### Molecular networking-based overview of the KP337 dataset

**Fig. 3:**
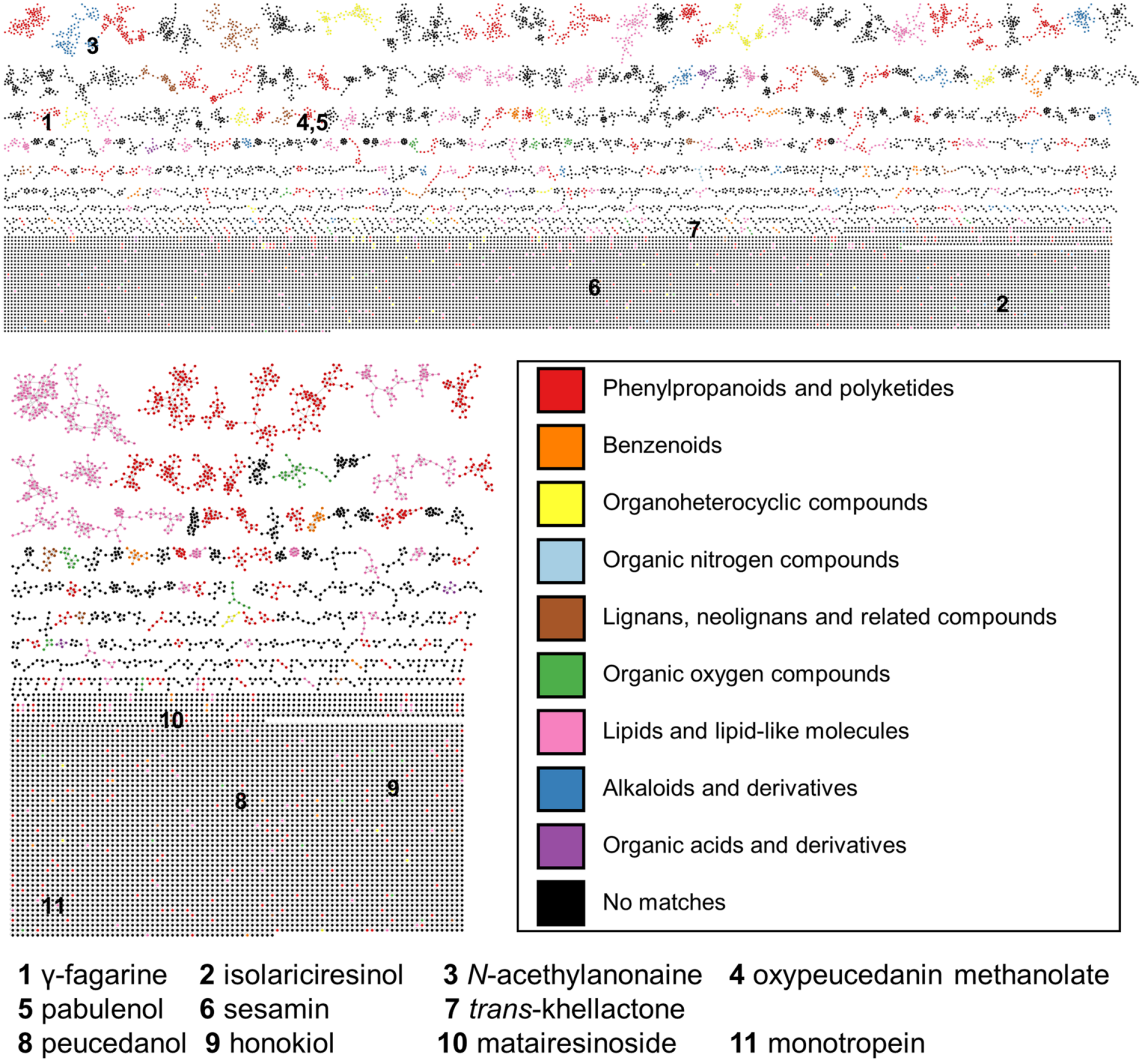
The molecular networks of the 337 medicinal plant samples, acquired in the positive (up) and negative (down) ion mode. The colours of spectral nodes denote class annotations given by the MolNetEnhancer workflow. Spectral nodes matched to the SSK legacy spectral library are represented by numbering.

Classical molecular networking analyses were performed with the datasets acquired in positive and negative ion mode^18^, respectively, to provide an overview of the specialized metabolome of the analyzed medicinal plants. The positive ion mode data yielded a molecular network consisting of 16,533 spectral nodes, while the negative ion mode data gave a network of 6,570 spectral nodes. The MolNetEnhancer workflow assigned class annotations to molecular families based on the spectral matching-based annotation^19^; the resulting molecular networks are shown in Fig. 3, and the class annotations are summarized in Table 1. In both networks, phenylpropanoids and polyketides account for the largest portion of the annotated molecular networks. This may be due to the high polyphenol content of the medicinal plants. Organic nitrogen compounds and alkaloids and derivatives were only observed in the positive ion mode data-based network because of the basicity of nitrogen-containing compounds. For the positive and negative ion mode networks, 81.6% (13,488 of 16,533) and 76.2% (5,005 of 6,570) of the spectral nodes were respectively unannotated. This can seem to be too low, but it needs to be denoted that the class of each molecular family was annotated only based on the spectral matching. The number of publicly available reference spectra are still much lower than the number of known phytochemicals; thus, application of *in silico* spectral annotation methods would increase the annotation rate, as we demonstrated in a previous study on specialized metabolome of the family Rhamnaceae^20^.

**Table 1:** Numbers of spectral nodes for each superclass-level annotation provided by the MolNetEnhancer workflow.

ClassyFire Superclass	Positive ion mode	Negative ion mode
Phenylpropanoids and polyketides	1274 (7.71%)	799 (12.16%)
Benzenoids	106 (0.64%)	44 (0.67%)
Organoheterocyclic compounds	266 (1.61%)	12 (0.18%)
Organic nitrogen compounds	6 (0.04%)	0 (0.00%)
Lignans, neolignans, and related compounds	221 (1.34%)	29 (0.44%)
Organic oxygen compounds	59 (0.36%)	67 (1.02%)
Lipids and lipid-like molecules	765 (4.63%)	602 (9.16%)
Alkaloids and derivatives	284 (1.72%)	0 (0.00%)
Organic acids and derivatives	64 (0.39%)	12 (0.18%)
No matches	13488 (81.58%)	5005 (76.18%)
Sum	16533	6570

### Spectroscopic validation of the phytochemicals

The structures of the purified phytochemicals were confirmed by manual inspection of the nuclear magnetic resonance (NMR) and MS spectra.

### Dereplication of the KP medicinal plants data against the spectral library

For further validation of the spectral library, we re-established the molecular networks of the KP337 dataset^18^ using the SSK legacy spectral library, together with all the spectral libraries available in GNPS. Consequently, 11 compounds (7 in positive and 4 in negative ion mode) were matched as the candidates with the highest scores (Table 2). Most of the sample occurrences in the matched spectra correlated with the previous reports of the matched molecules from taxonomically same or close species, which supported the reliability of the dereplication result. γ-Fagarine was isolated from *Phellodendron amurense*^21^ and *Dictamnus albus*^22^, while sesamin was found in *Asarum heterotropoides*^23^, and oxypeucedanin methanolate and pabulenol were isolated from *Angelica dahurica*^24,25^. Isolariciresinol, which was reported from *Rubia argyi*^26^, has not previously been reported in *Patrinia scabiosifolia*; however, it was reported from *P. scabra*, another species of the genus^27^. Due to the possible conservation of biosynthesis in close taxa, species in the same genus or family often contain the same or similar specialized metabolites^28^. Thus, the occurrence of isolacriciresnol in *P. scabiosifolia* can be supported by the occurrence of the same compound in *P. scabra*. Spectral matching suggested the occurrence of *trans*-khellactone and peucedanol in *Glehnia littoralis*, but neither of these two compounds have been reported in the plant. However, six *O*-acyl derivatives of *cis-*khellactone were reported from *G. littoralis*^29^. Along with the spectral matching results, this suggests the occurrence of *cis*-khellactone in *G. littoralis*, as the *cis*/*trans* isomers cannot be distinguished by MS/MS analysis. Similarly, oxypeucedanol has not been reported in *G. littoralis*, but multiple oxypeucedanol glycosides were reported from *G. littoralis*, which supports the occurrence of the aglycone in this plant^30^. These cases simultaneously highlight the applicability and value of the dataset and spectral library introduced in this Data Descriptor; the coverage of spectral matching was expanded, and the occurrence of previously known compounds can be easily estimated by searching the spectra against the dataset.

**Table 2:** Spectral matches between KP337 dataset and KSS legacy library.

Compound	Ion mode	Cosine	Occurrence	Comment
γ-fagarine	positive	0.98	*Phellodendron amurense* (cortex)	Described from *P. amurense*^21^
			*Dictamnus albus* (cortex)	Described from *D. albus*^22^
isolariciresinol	positive	0.85	*Patrinia scabiosifolia* (root)	Described from other *Patrinia* species^27^
			*Rubia argyi* (root)	Described from *R. argyi*^26^
*N*-acetylanonaine	positive	0.71	*Nelumbo nucifera* (whole/root)	Analogues are described from *N. nucifera*^31^
oxypeucedanin methanolate	positive	0.81	*Angelica dahurica* (root)	Described from *A. dahurica*^24,25^
pabulenol	positive	0.84	*Angelica dahurica* (root)	Described from *A. dahurica*^24,25^
sesamin	positive	0.79	*Asarum heterotropoides* (root)	Described from *A. heterotropoides*^23^
*trans*-khellactone	positive	0.76	*Glehnia littoralis* (root)	Analogues are described from *G. littoralis*^29^
peucedanol	negative	0.85	*Peucedanum japonicum* (root)	Described from *P. japonicum*^32^
			*Peucedanum praeruptorum* (root)	Described from *P. praeruptorum*^33^
			*Glehnia littoralis* (root)	Analogues are described from *G. littoralis*^30^
honokiol	negative	0.93	*Magnolia obovata* (cortex)	Described from *M. obovata*^34^
matairesinoside	negative	0.66	*Trachelospermum asiaticum* (whole)	Described from *T. asiaticum*^35^
			*Arctium lappa* (seed)	Described from *A. lappa*^36^
			*Patrinia scabiosifolia* (root)	—
			*Forsythia suspensa* (fruit)	Described from *F. suspensa*^37^
Monotropein	negative	0.67	*Pyrola japonica* (whole)	Described from *P. japonica*^38^
			*Gynochthodes officinalis* (root)	—

## Supplementary information

Supplementary Table 1

## Data Availability

The data processing workflow for establishing the spectral library is available in GNPS.
